# Clinical and morphological characteristics of patients with idiopathic epiretinal membrane with foveal herniation

**DOI:** 10.1038/s41433-022-02094-3

**Published:** 2022-06-13

**Authors:** Mehmet Murat Uzel, Faik Gelisken, Eva Konrad, Jonas Neubauer

**Affiliations:** 1 Department of Ophthalmology, Balıkesir University School of Medicine, Balıkesir, Turkey; 2 Department of Ophthalmology, Eberhard Karls University, Tübingen, Germany

**Keywords:** Outcomes research, Retinal diseases, Risk factors

## Abstract

**Purpose:**

To analyse the clinical and morphological characteristics of eyes with idiopathic epiretinal membrane (iERM) and foveal herniation (FH).

**Methods:**

Clinical findings and OCT features of patients with iERM and FH were retrospectively analysed. Primary outcome were changes of the best-corrected-visual-acuity (BCVA) and OCT features from baseline to the last visit. FH patients were divided into two groups based on herniated layers: ganglion cell complex (GCC)-group and sub-GCC-group. Surgical outcome was also assessed.

**Results:**

In this study, 3882 patients with iERM were screened, of whom 51 (1.3%) were identified with FH. The GCC-group (*n* = 16) had a better baseline BCVA and thinner central foveal thickness (CFT) in comparison to the sub-GCC-group (*n* = 35) but without statistical significance (*p* = 0.330, *p* = 0.417, respectively). The postoperative BCVA-improvement was similar between the two groups (*p* = 0.280). Fibrillary surface changes were detected in 42/51 (82.3%) patients, significantly more often in the sub-GCC group (*p* = 0.020). The baseline BCVA was a predictive factor for the postoperative vision change.

**Conclusion:**

FH presents with a unique macular morphology in eyes with iERM. Affected eyes experience varying visual disturbances based on the involvement of the inner retinal layers in the foveal herniation. Macular surgery is successful in restoring vision, even though foveal morphology does not fully recover.

## Introduction

Idiopathic epiretinal membrane (iERM) is a disease characterized by the development of an avascular fibrocellular membrane on the surface of the internal limiting membrane (ILM) [1]. The incidence of iERM has been reported to be between 5% and 28%, increasing with older age [2, 3]. Tangential traction of the ERM may cause structural changes in the macula, especially in the inner retinal layers, leading to impairment of vision [4].

Because of recent progress in optical coherence tomography (OCT) imaging and experience in interpretation, numerous morphological features associated with iERM were reported, such as tractional changes on central bouquet, cotton ball sign (CB), fibrillary surface changes (FSC), ectopic inner foveal layers and foveal herniation (FH) [5–9].

FH in iERM was first reported in 2011 by Francis et al. as a perifoveal, circumferential contraction of the ERM and the subsequent prolapse of the foveola centrally [10]. Thereafter, only a few studies reported on FH in iERM [11–15]. In the presented study we analyse the clinical and morphological characteristics of FH in a large cohort of patients with iERM.

## Methods

In this retrospective study, all consecutive patients with the diagnosis of iERM at the Ophthalmology Department of University of Tuebingen from March 2008 till December 2020 were screened. The study adhered to the Declaration of Helsinki and was approved by the institutional ethics committee at the university of Tuebingen.

FH was identified by an experienced physician (FG) on OCT scans. Exclusion criteria were: (1) Presence of vitreomacular traction, (2) Secondary ERM, (3) Previous ocular surgery except for phacoemulsification, (4) Poor image quality of OCT, (5) Myopia >−6 Dioptre, (6) Coexistence of retinal diseases that could have an impact on the macular morphology such as diabetic retinopathy, uveitis or retinal vein occlusion.

Demographic characteristics of the patients, best-corrected visual acuity (BCVA) determined by Snellen chart, status of the fellow eye, follow-up time before and after the surgery and type of the intraocular tamponade was recorded. Besides the FH, central foveal thickness (CFT), FSC, intraretinal cystoid space, CB and integrity of the ellipsoid zone (EZ) were recorded. FH was defined as displacement of retinal tissues through the opening of the ERM in the foveal region into the vitreous space [10, 15]. CFT was defined as the distance between the inner retinal surface and the inner border of the retinal pigment epithelium and measured with the software provided by Spectralis SD OCT (Heidelberg Eye Explorer version 1.9.13.0, Spectralis Viewing Module 6.5.2.0, Heidelberg Engineering, Germany). If necessary, the generated segmentation lines were manually corrected. CB was present if a thickened roundish fuzzy hyperreflective area between the inner segment-outer segment and cone outer segment tip line was seen in the fovea center [5, 6]. Intraretinal cystoid spaces were defined as the presence of hyporeflective small, round lesions in the retina. FSC was described as hypo- and hyperreflective parallel stripes between over or above the retinal nerve fiber layer and ERM or ILM [7, 8]. EZ integrity was classified as intact, partially disrupted and totally disrupted, based on its visibility on OCT examination [16]. The parameters were recorded at baseline and at the last visit. Additionally, for the eyes that underwent a surgical treatment, the preoperative findings were compared with the last postoperative visit.

**Fig. 1:**
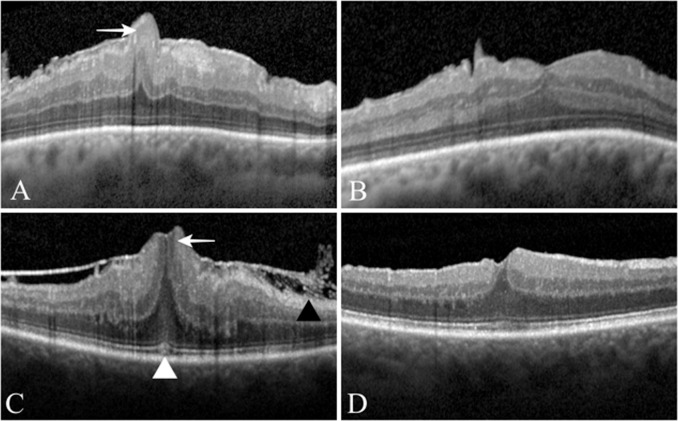
Preoperative and postoperative OCT images of an eye with epiretinal membrane and foveal herniation. **A** Herniation of ganglion cell complex (GCC) (white arrow). **B** Three months after surgery, foveal contour is restored and there are focal epiretinal folds, parafoveally. **C** Herniation of sub-ganglion cell complex (sub-GCC) retinal layers (white arrow), cotton ball sign (white arrow head) and fibrillary surface changes (black arrow head). **D** Foveal contour is partially restored 12 months after surgery.

Patients were divided into two groups. Ganglion cell complex group (GCC) presented with the herniation of the three innermost retinal layers, namely the nerve fibre, ganglion cell and inner plexiform layers, whereas in sub-ganglion cell complex group (sub-GCC) included eyes with involvement of additional retinal layers to the herniation (Fig. 1).

Macular surgery included a 23-gauge pars plana vitrectomy (Qube Pro, Fritz Ruck Ophthalmologische Systeme GmbH, Germany) and a peeling of all epiretinal membranes and ILM using a dye (Membraneblue-Dual, DORC, The Netherlands).

Descriptive and statistical analysis was performed using IBM SPSS Statistics 21. Demographic characteristics were expressed as mean, standard deviation, frequency or percentage according to clinical and OCT findings. Visual acuity was converted to the logarithm of the Minimum Angle of Resolution (logMAR) for statistical analysis. Baseline BCVA and changes in BCVA were analysed after excluding three patients with dense cataracts. The normal distribution test of continuous data was evaluated with the Kolmogorov–Smirnov test. Since the data conformed to normal distribution, independent *t*-test was used in independent groups and paired *t*-test was used in dependent groups. Categorical data were evaluated using the chi-square test. Factors affecting baseline BCVA, postoperative BCVA change and anatomical outcomes were tested using a linear or binary logistic regression model. Statistical significance was accepted with a *p* value of 0.05 or less.

## Results

From March 1, 2008 to December 31, 2020, 3882 patients were diagnosed with iERM. After analysing the OCT images, FH was identified in 51 eyes of 51 patients (1.3%). The mean age of the patients with FH was 72.67 ± 7.11 years (range 57–91 years) with 27 (52.9%) of them being female. Baseline BCVA was 0.55 ± 0.21 logMAR (Snellen equivalent 20/70) and baseline CFT was 677.14 ± 97.36 µm. Twenty-one (41.1%) eyes had intraretinal cystoid space, 10 (19.6%) eyes CB, 42 (82.3%) eyes FSC and three (5.8%) eyes partial disrupted EZ. Baseline characteristics according to the groups are given in Table 1. All additional OCT features were more common in the sub-GCC group, however only the presence of FSC reached a statistical significance (*p* = 0.02).

**Table 1:** Baseline characteristics of the study population.

	GCC group *n* = 16	Sub-GCC group *n* = 35	*p* value
Age (years, mean ± SD)	71.38 ± 7.39	73.26	0.386
BCVA^a^ (logMAR, mean ± SD)	0.53 ± 0.19	0.56 ± 0.22	0.670
CFT (µm, mean ± SD)	660.56 ± 89.26	684.71 ± 101.18	0.417
IRP, *n* (%)	4 (25)	17 (48.6)	0.137
CB, *n* (%)	3 (18.7)	7 (20)	0.917
FSC, *n* (%)	10 (62.5)	32 (91.4)	0.020
EZ integrity (partial disrupted) *n* (%)	–	3 (8.6)	–

*SD* standard deviation, *BCVA* best corrected visual acuity, *CB* cotton ball sign, *FSC* fibrillary surface changes, *EZ* elipsoid zone, *GCC* ganglion cell complex, *sub-GCC* sub-ganglion cell complex, *logMAR* logarithm of the minimum angle of resolution, *CFT* central foveal thickness, *IRP* intraretinal pseudocyst.

^a^Three patients with cataracts were excluded from sub-GCC group.

Forty-three (84.3%) of the 51 eyes underwent surgical treatment. In three eyes, the vitrectomy was combined with phacoemulsification. Intraocular gas tamponade was used at the surgeon’s discretion. In seven eyes (16.2%) intraocular gas (four eyes C2F6 and three eyes air) tamponade was given and the remaining 36 eyes (83.7%) were left with balanced salt solution. Eight (18.6%) eyes developed cataracts and were operated later. Retinal detachment was noted in one, recurrent ERM in two and postoperative macular oedema in one eye.

Thirty-one (72%) of the 43 operated patients with a postoperative follow-up for at least three months were evaluated. The mean postoperative follow-up time was 35.90 ± 38.06 months (range 3–130 months, median 17 months). The preoperative BCVA of the patients was 0.56 ± 0.21 logMAR (Snellen equivalent 20/72) and improved to 0.25 ± 0.17 logMAR (Snellen equivalent 20/35) (*p* < 0.001) at the final visit. The average improvement of the BCVA was found in GCC and sub-GCC groups as 0.28 ± 0.13 and 0.32 ± 0.19 logMAR (*p* = 0.499), respectively. Preoperative CFT was 682.06 ± 96.49 µm and decreased postoperatively to 407.26 ± 78.75 µm (*p* < 0.001) at the final visit. The average decrease in CFT in the GCC and sub-GCC groups was 264.81 ± 101.93 µm and 280.30 ± 124.17 µm, respectively. The baseline BCVA was a predictive factor for the change in BCVA after the surgery (R2 0.369, ß coefficient 0.484, *p* = 0.001).

Foveal contour remodelling was normal or shallow in four of seven eyes with gas tamponade and only in seven of 24 patients without gas tamponade (*p* = 0.210). There was no statistically significant difference between the change of foveal contour and vision (*p* = 0.488). The use of tamponade and foveal contour changes according to the groups are given in Table 2.

**Table 2:** Intraocular tamponade and change of the foveal contour.

	GCC group, *n* = 11		Sub-GCC group, *n* = 20	
Foveal contour	with tamponade	without tamponade	with tamponade	without tamponade
Normal, *n* (%)				1 (5)
Shallow, *n* (%)	1 (9.1)	3 (27.3)	3 (15)	3 (15)
Flat, *n* (%)	1 (9.1)	2 (18.2)	1 (5)	4 (20)
Convex, *n* (%)	1 (9.1)	3 (27.3)		8 (40)

*GCC* ganglion cell complex, *Sub-GCC* sub-ganglion cell complex.

Age and CFT were found to be associated with the baseline BCVA by multivariable linear regression analysis (R2 0.336, ß coefficient 0.017, *p* < 0.001, ß coefficient 0.001, *p* = 0.005, respectively). No other baseline clinical parameter or OCT features were found to be associated with the postoperative change of the CFT.

## Discussion

The presented study revealed that FH is a rare feature in iERM with an incidence of 1.3% in our cohort. It was seen mostly in the elderly population without a gender predilection. The most common accompanying OCT finding was FSC, seen frequently in the sub-GCC group, followed by intraretinal cystoid spaces. Macular surgery was effective in improvement of the vision and decrease of thickened CFT.

FH was first reported at the ARVO meeting in 2011 by Francis et al. [10] in six eyes. Histopathological examination of the surgical excised ERM in these eyes revealed smooth muscle actin and fibrocellular membranes consistent with metaplastic RPE cells [10].

In 2017, Ozdemir and Karacorlu reported a case with FH in an eye with ERM [15]. A multicentre, retrospective study by Ozdek et al. and a retrospective case series by Ozkaya et al. analysed the surgical outcome of the eyes with iERM and among them FH [12, 13]. The incidence of FH were reported as 20.1% in 634 eyes that underwent a macular surgery for iERM [12]. In the present study, FH rate was 1.3%. This discrepancy is more likely because of the difference in the composition of the cohorts. Our study population included all eyes diagnosed with iERM, whereas the other cohort consisted only of eyes that underwent macular surgery. Another possible reason could be differences in the interobserver rates for interpretation of OCT-images in the setting of a multicentre study design. Similar to other reports, a visual gain was found in our study with an average of three lines after the macular surgery. Restoration of the foveal contour was achieved in 35.5%, which was comparable to previous studies reporting between 32.2% and 45.4% of successful restoration [12, 13]. In our population, eyes having intraocular gas (16.2%) had higher chance of restoration of the foveal contour. Intraocular gas tamponade was used more frequently in other studies (54.5–86%) than in our study [12, 13]. No correlation between the improvement of the vision and the use of tamponade or restoration of the foveal contour was found. On the other hand, the restoration of the fovea contour may contribute to the improvement of the distorted vision that was not addressed in the presented study. In our study, we found that the lower the baseline BCVA, the greater was the postoperative vision increase. This may suggest that patients could benefit significantly from macular surgery even though they present with poor vision initially.

In the present study, eyes were classified into two groups in order to find a possible link between the involvement of the retinal layers in FH and the severity of the morphological and functional status of the macula. Baseline BCVA was lower and CFT higher in the sub-GCC group, however, without reaching a level of significance. The most common finding accompanying FH was FSCs observed more frequently in the sub-GCC group. In addition, the presence of intraretinal cystoid spaces, CB, partially disrupted EZ were higher in the sub-GCC group. It is reported that FCS causes greater adhesion of ERM and increases surgical difficulty [7, 8]. CB is thought to be a result of the transfer of retinal surface traction to the foveal central bouquet.

The exact mechanisms of the FH are not known. FH may occur as a result of chronic tangential traction in eyes with ERM. It was shown that ILM is the thinnest in the centre of the fovea at 400 nm [17]. Therefore, it is more likely that retinal tissue herniates from this part of the fovea. The presence of fibrillary surface change and tractioned central bouquet changes, frequently seen in FH are other possible signs, indicating the chronicity and severity of the traction on the surface of the macula in these eyes.

There are limitations of our study due to its retrospective design. Not all patients were followed up in a comparable manner and the lens status differed, so time-dependent changes in visual acuity could not be precisely documented. In addition, although over 3800 patients were analysed, we only found a rather small number of cases with FH to be investigated. This, however, was due to the low frequency of this entity.

In conclusion, FH presents with a unique foveal morphology in iERM patients. It is often associated with tractional findings such as fibrillary surface changes and tractional central bouquet changes. Although the foveal anatomy does not improve in every patient after macular surgery, functional improvement is achieved to a greater extent. Further reports will clarify the role of therapeutic measures such as the impact of ILM peeling, use of intraocular tamponade as well as the role of the subgroups in patients with iERM and FH.

### Summary

#### What was known before

- Idiopathic epiretinal membrane can lead to tangential traction of the retina and therefore may cause structural changes in the macula, especially in the inner retinal layers, leading to impairment of vision.
- Foveal Herniation was first described 2011 and is formed as a result of prolapse of the foveola centrally, peripheral contraction and folding of the inner retina.

#### What this study adds

- 3882 patients with iERM where screened, of whom 51 (1,3%) were identified with Foveal herniation.
- Foveal herniation patients were divided into two groups based on herniated layers: ganglion cell complex (GCC)-group and sub-GCC-group and it was shown, that the sub-GCC group had more severely affected macular morphology.
- The baseline BCVA was a predictive factor for the postoperative vision change.

## Data Availability

Most of the data generated or analysed during this study are included in this published article. Further data are not publicly available due to data privacy reasons but are available from the corresponding author on reasonable request.
